# Nisin in Combination with Cinnamaldehyde and EDTA to Control Growth of *Escherichia coli* Strains of Swine Origin

**DOI:** 10.3390/antibiotics6040035

**Published:** 2017-12-12

**Authors:** Des Field, Inès Baghou, Mary C. Rea, Gillian E. Gardiner, R. Paul Ross, Colin Hill

**Affiliations:** 1 School of Microbiology, University College Cork, Cork T12 YT20, Ireland; ines.baghou@mycit.ie (I.B.); p.ross@ucc.ie (R.P.R.); 2 Teagasc Food Research Centre, Moorepark, Fermoy, Co., Cork P61 C996, Ireland; mary.rea@teagasc.ie; 3 APC Microbiome Institute, University College Cork, Cork T12 YT20, Ireland; 4 Department of Science, Waterford Institute of Technology, Waterford X91 K0EK, Ireland; ggardiner@wit.ie

**Keywords:** antimicrobial resistance, antibiotics, antimicrobial peptide, enterotoxigenic *E. coli*, nisin, bacteriocin, essential oil, cinnamaldehyde, EDTA

## Abstract

Post-weaning diarrhoea (PWD) due to enterotoxigenic *Escherichia coli* (ETEC) is an economically important disease in pig production worldwide. Although antibiotics have contributed significantly to mitigate the economic losses caused by PWD, there is major concern over the increased incidence of antimicrobial resistance among bacteria isolated from pigs. Consequently, suitable alternatives that are safe and effective are urgently required. Many naturally occurring compounds, including the antimicrobial peptide nisin and a number of plant essential oils, have been widely studied and are reported to be effective as antimicrobial agents against pathogenic microorganisms. Here, we evaluate the potential of nisin in combination with the essential oil cinnamaldehyde and ethylenediaminetetraacetic acid (EDTA) to control the growth of *E. coli* strains of swine origin including two characterized as ETEC. The results reveal that the use of nisin (10 μM) with low concentrations of trans-cinnamaldehyde (125 μg/mL) and EDTA (0.25–2%) resulted in extended lag phases of growth compared to when either antimicrobial is used alone. Further analysis through kill curves revealed that an approximate 1-log reduction in *E. coli* cell counts was observed against the majority of targets tested following 3 h incubation. These results highlight the potential benefits of combining the natural antimicrobial nisin with trans-cinnamaldehyde and EDTA as a new approach for the inhibition of *E. coli* strains of swine origin.

## 1. Introduction

Post-weaning diarrhea (PWD) is one of the most life-threatening diseases in the swine industry worldwide. It is commonly associated with the proliferation of enterotoxigenic *Escherichia coli* (ETEC) in the pig intestine resulting in mortality, dehydration, weight loss and retarded growth [1]. Current control strategies frequently involve polymyxins, macrolides and fluoroquinolones, antibiotics that are also critically important in human medicine [2]. However, the routine use of in-feed antibiotics was banned in the European Union (EU) in 2006 (Regulation EC/1831/2003), although their use is still permitted under veterinary prescription as the need arises. Moreover, in recent times the use of antimicrobials in food production animals has come under considerable scrutiny, given that recent genomic and metagenomic studies in humans, animals and in the environment have brought to light the existence of a reservoir of antibiotic resistance genes that could be mobilized and transferred from these sources to human pathogens [3,4,5]. In addition, the use of zinc oxide (ZnO) at pharmacological concentrations (i.e., concentrations in excess of normal dietary requirements) will no longer be a therapeutic option for the prevention and control of PWD and bowel oedema in young pigs in the EU. This follows the recent ruling by the Committee for Medicinal Products for Veterinary Use (CVMP) to ban its use. Consequently, there is increasing emphasis on practices to reduce antibiotic usage in animal husbandry by promoting prudent use initiatives, as well as exploring the implementation of potential alternatives for the use of antibiotics and zinc oxide in livestock production.

One group of antimicrobials that have been at the forefront of alternative antibiotic research for decades are the lantibiotic class of bacteriocins (bacterially produced, small, heat-stable peptides that are active against other bacteria). The best known lantibiotic is nisin A, a 34-amino acid polycyclic peptide that exhibits antibacterial activity against a wide range of clinical and food-borne pathogens and is widely used as a natural food biopreservative [6]. Although nisin is not currently used for medical applications, it has realized commercial applications in the veterinary industry for the prevention/treatment of bovine mastitis [7,8]. It functions by a distinctive dual mode of action involving binding to lipid II, an essential precursor of the bacterial cell wall, followed by insertion into the membrane of the target cell to form a pore [9,10,11]. As a consequence of these two distinct and cooperative mechanisms, no significant resistance to nisin A has been observed despite its widespread use over several decades by the food industry [12]. Consequently, lantibiotics such as nisin A possess enormous potential for therapeutic applications, not only as alternatives but also as synergists with other antimicrobials. For example, aromatic plant oils have been widely investigated due to their antimicrobial activities and numerous studies have demonstrated the synergistic activities of nisin and essential oil combinations including thymol [13], carvacrol [14,15] and cinnamaldehyde [16], amongst others. Similarly, the activity of nisin can be enhanced through the addition of chelating agents such as ethylenediaminetetraacetic acid (EDTA) [17,18]. Recently, Al Atya and coworkers demonstrated the effectiveness of nisin in combination with colistin towards *E. coli* strains of swine origin [19]. Importantly, the combination proved effective against both planktonic and biofilm cultures of *E. coli* strains exhibiting a colistin-resistance phenotype, validating the potential of such strategies to reduce the effective dose required for these antibiotics to help prevent or delay the further spread of resistance. Indeed, such approaches are all the more urgent given the directive that all EU Member States are required to achieve a reduction of approximately 65% in the current sales of colistin for veterinary use at an EU level by 2020 (https://www.cdc.gov/drugresistance/tatfar/tatfar-recomendations.html). However, if the use of colistin and other critical antibiotics is to be diminished or replaced in animal husbandry, suitable alternatives that are safe and efficient must be found.

Here we assess the efficacy of nisin in combination with a variety of essential oils and EDTA, and establish that a nisin + EDTA + cinnamaldehyde combination exhibits significantly greater anti-*E coli* activity compared to the use of either antimicrobial alone.

## 2. Results

### 2.1. Bacterial Susceptibility to Antimicrobial Compounds

Minimum inhibitory concentration assays (MICs) with purified nisin A peptide and the essential oils thymol, carvacrol and trans-cinnamaldehyde were determined against *E. coli* targets of pig origin, including two characterized as ETEC (K88F4 and F18ab) in order to ascertain appropriate concentrations for combinatorial assays. Additionally, MIC assays were also carried out to establish the relative sensitivity or resistance to a range of antibiotics including penicillin, erythromycin, chloramphenicol, tetracycline, cefuroxime, ceftazidime, colistin and polymyxin B. Activity against the target strains required a relatively high concentration of nisin (200 µg/mL). This value was in agreement with data obtained by Naghmouchi and co-workers against a panel of Gram-negative strains [20] and, yet again, highlights the relative resistance of Gram-negative bacteria to nisin compared to Gram-positive strains which can have MICs in the nanomolar (nM) range. The majority of the *E. coli* targets were resistant to erythromycin, tetracycline and penicillin, with the exception of *E. coli* F2S2, which remained sensitive to chloramphenicol and tetracycline (Table 1) and were overall in agreement with previous studies with *E. coli* strains of swine origin [19,21]. Colistin and polymyxin B exhibited almost identical activity against 4 of 5 of the target strains and were in close agreement with previously established figures against sensitive strains of *E. coli* [19,22]. The exception was the ETEC F18ab strain which exhibited an MIC of 25 µg/mL for both polymyxin and colistin (Table 1), indicative of resistance to these antimicrobials. Furthermore, all of the target strains remained sensitive to the cephalosporin antibiotics ceftazidime, cefuroxime and cefradine, while resistance to cefoxitin was observed for all strains. The susceptibility of the bacterial strains to the essential oils thymol, carvacrol and trans-cinnamaldehyde was also assessed in order to ascertain appropriate concentrations for combinatorial assays. Cinnamaldehyde proved to be the most active of the essential oils with an inhibitory concentration of 1250 μg mL^−1^ which was within the 400–1322 μg mL^−1^ range established by previous studies [23].

### 2.2. Growth Curve-Based Comparisons of the Activity of Nisin A and Natural Antimicrobial Combinations

**Figure 1:**
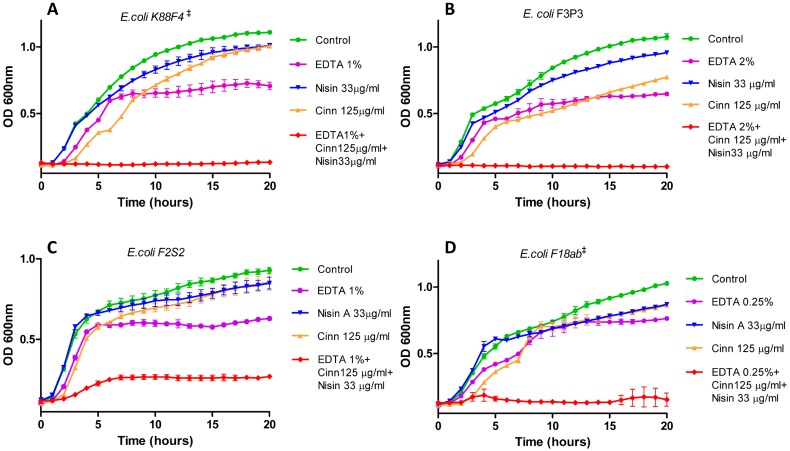
Growth curve analysis of (**A**) *E. coli* K88F4 in the presence of 1/6× minimum inhibitory concentration (MIC; 33 μg/mL) nisin A (blue inverted triangle), 1/10× MIC (125 μg/mL) cinnamaldehyde (orange triangle), ethylenediaminetetraacetic acid (EDTA) 1.0% (purple circle) in combination (red diamond) and untreated control (green circle), (**B**) *E. coli* F3P3 in the presence of 1/6× MIC (33 μg/mL) nisin A (blue inverted triangle), 1/10× MIC (125 μg/mL) cinnamaldehyde (orange triangle), EDTA 2% (purple circle), in combination (red diamond) and untreated control (green circle), (**C**) *E. coli* F2S2 in the presence of 1/6× MIC (33 μg/mL) nisin A (blue inverted triangle), 1/10× MIC (125 μg/mL) cinnamaldehyde (orange triangle), EDTA 1% (purple circle), in combination (red diamond) and untreated control (green circle) and (**D**) *E. coli* F18ab in the presence of 1/6× MIC (33 μg/mL) nisin A (blue inverted triangle), 1/10× MIC (125 μg/mL) cinnamaldehyde (orange triangle), EDTA 0.25% (purple circle), in combination (red diamond) and untreated control (green circle). ^‡^ denotes ETEC strains.

Having established the MIC values for nisin A and a range of essential oils against the panel of *E. coli* strains, growth curves were performed in order to reveal the impact of sub-lethal concentrations of nisin A, cinnamaldehyde and EDTA (alone and in combination) on bacterial growth. The final concentration of nisin or cinnamaldehyde used for each organism was a fraction of the previously determined MIC value (i.e., 1/10×, 1/6×, 1/4×, etc.) and combinations thereof. Initial growth curves were carried out with EDTA to establish levels that were marginally inhibitory compared to a control strain in the absence of EDTA for each of the strains. Nisin at 1/6× MIC (33 μg/mL) had little or no impact on the growth of the targets *E. coli* K88F4 (Figure 1A), *E. coli* F3P3 (Figure 1B) or *E. coli* F2S2 (Figure 1C), affirming the relatively poor activity of nisin against Gram-negative bacteria. Similarly, a slight lag in growth was observed for both EDTA and cinnamaldehyde (at 1/10× MIC; 125 μg/mL) when compared to the untreated control (Figure 1). However, pronounced inhibitory effects were recorded when nisin was used in combination with cinnamaldehyde and EDTA against *E. coli* K88F4 (Figure 1A), *E. coli* F3P3 (Figure 1B), *E. coli* F2S2 (Figure 1C), as observed by the exceptionally extended lag phase in all cases compared to the untreated control. Notably, the combination also proved effective against the colistin and polymyxin B-resistant ETEC F18ab strain (Figure 1D).

### 2.3. Kill Assay Determination of the Activity of Nisin A and Natural Antimicrobial Combinations

Following on from the data established by growth curves, the bactericidal activity of nisin in combination with cinnamaldehyde and EDTA against the target *E. coli* was investigated utilising kill assays within a defined period of time (3 h). *E. coli* F2S2, *E. coli* K88F4, *E. coli* F3P3 and *E. coli* F18ab at a concentration of 1 × 10^7^ cfu were exposed to nisin (33 μg/mL), cinnamaldehyde (125 μg/mL) and EDTA (1%, 2%, 2%, 0.25%, respectively) at 37 °C for 3 h.

**Figure 2:**
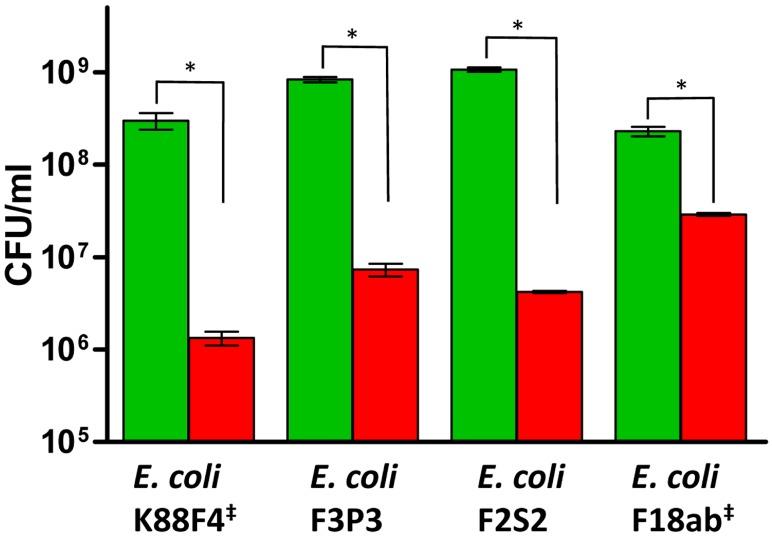
Kill effect of nisin in combination with cinnamaldehyde and EDTA against *E. coli* strains of swine origin. Kill assay performed over a defined 3 h period with *E. coli* K88F4 in the presence of nisin (33 μg/mL) + cinnamaldehyde (125 μg/mL) + EDTA (1%) and untreated control (Green), *E. coli* F3P3 in the presence of nisin (33 μg/mL) + cinnamaldehyde (125 μg/mL) + EDTA (2%) (Red) and untreated control (Green), *E. coli* F2S2 in the presence of nisin (33 μg/mL) + cinnamaldehyde (125 μg/mL) + EDTA (1%) EDTA 2% (Red) and untreated control (Green), *E. coli* F18ab in the presence of nisin (33 μg/mL) + cinnamaldehyde (125 μg/mL) + EDTA (0.25%) (Red) and untreated control (Green). Asterisks rating of * indicates statistically significant differences between groups (*p* ≤ 0.05). ^‡^ denotes ETEC strains.

In general, an approximate 1-log reduction in *E. coli* counts was observed for the antimicrobial combination compared to the initial inoculum (Figure 2). When compared to the untreated control, a 2-log differential in cell numbers was observed between the antimicrobial combination and the control in each case for *E. coli* K88F4, (*p* < 0.046), *E. coli* F3P3 (*p* < 0.043) and *E. coli* F2S2 (*p* < 0.005). The exception was *E. coli* F18ab whereby cell numbers remained static (2.9 × 10^7^ cfu/mL; Figure 2) following 3 h of incubation. In contrast, the untreated control had increased to 2.3 × 10^8^ cfu/mL.

## 3. Discussion

Post-weaning diarrhea is an economically important enteric disease in pigs resulting in significant financial losses. It is commonly associated with the proliferation of ETEC, a pathotype characterized by the production of enterotoxins and adhesins, both essential for disease development [24] that act on the intestinal epithelium of pigs. Alarmingly, critically important antibiotics such as fluoroquinolones, aminoglycosides and polymyxins are sold in vast quantities for use in pigs during the post-weaning period in many countries worldwide [25]. However, the emergence of bacterial resistance to these antibiotics has dictated that effective and sustainable alternative approaches to tackling microbial disease in both humans and livestock must be identified. Here, we set out to examine for the first time the ability of nisin, when used in conjunction with a selection of natural antimicrobials including essential oils, to control several *E. coli* strains of swine origin, including two characterized as ETEC, with the ultimate aim of identifying superior antimicrobial combinations. Following MIC determinations and growth curve analysis in the presence of nisin + cinnamaldehyde + EDTA combinations, substantial enhanced inhibitory relationships were observed. The results reveal that sub-inhibitory levels of nisin (1/6× MIC) and cinnamaldehyde (1/10× MIC) supplemented with EDTA (0.25–2%) can effectively control the growth of *E. coli* numbers in laboratory media. In contrast, nisin was ineffective against the *E. coli* targets when used independently. Indeed, considerable work has been conducted to enhance the poor antibacterial activity of nisin toward Gram-negative bacteria (due to the outer membrane of the Gram-negative cell wall that acts as a physical barrier, obstructing access of the peptides to the cytoplasmic membrane). Such strategies have included destabilization of the outer membrane by combining with essential oils that contain aldehydes, terpenes and phenolic compounds [26,27]. Other strategies have involved nisin and chelating agents such as EDTA, which acts by removing Mg^2+^ and Ca^2+^ ions essential for stabilizing the lipopolysaccharide (LPS) outer membrane of Gram-negative bacteria [18,28]. Our investigations also highlight the enhanced potency of nisin when combined with trans-cinnamaldehyde and EDTA, implying that combinations of two or more antimicrobials, which affect different targets, exhibit great potential as a new approach against pathogenic-resistant bacteria. Moreover, all the aforementioned antimicrobials are classified as GRAS (Generally Recognized as Safe) by the FDA (Food and Drug Administration). Notably, a product containing the sodium salt of EDTA (Na_2_EDTA), a tannin-rich extract of *Castanea sativa*, thyme oil and oregano oil was recently assessed by EFSA for use with pigs at a recommended dose of 1000 mg/kg feed to reduce the incidence of dysentery caused by *Brachyspira hyodysenteriae* and so improve performance [29]. Furthermore, the European Food Safety Authority (EFSA) panel concluded that the additive was considered safe for pigs at the recommended dose of 1000 mg/kg feed of which Na_2_EDTA constituted 24% (equivalent to 240 mg). None of the active constituents raised safety concerns for consumers when considered individually and at the concentrations delivered to feed using the recommended dose. Indeed, animal feeding studies to investigate EDTA toxicity have established an LD_50_ value of 2000–2200 mg/kg bw for rats, while a no observed adverse effect level (NOAEL) of 500 mg/kg bw per day for Na_2_EDTA and Na_3_EDTA was determined from a 90-day study in rats and in a long-term (2-year) study in rats and mice [30]. However, rats fed 1%, 5% or 10% disodium salt of EDTA for 90 days had significantly lower food consumption and weight gain than controls [31]. Although the concentrations of EDTA used in this study (0.25–2%) exceed the maximum permitted dose (by 10–100 fold), reductions in EDTA concentration may be achieved through Response Surface Methodology which has been used very effectively to analyze, predict and model systems that require optimization of multiple factors and has been used previously for nisin, EDTA and pH combinations [32]. Alternatively, other chelators could be investigated as well as the use of other natural antimicrobial compounds such as organic acids, which have been shown to control PWD and enhance growth performance in pigs [33,34,35] and have been shown to act synergistically with nisin [36].

Several studies have investigated the ability of essential oil compounds to control the proliferation of pathogenic bacteria, as well as contribute to better gut health in pigs and potentially replace the use of antibiotic growth promoters which have been prohibited in the European Union since 2006 [37]. For example, Li and co-workers demonstrated that the addition of encapsulated essential oils (thymol and cinnamaldehyde) improved feed intake and growth rate, reduced the incidence of diarrhoea and resulted in a positive modulation of microbial populations measured in the faeces, with a reduction of *E. coli* and an increase of *Lactobacillus* counts [38]. Similarly, addition of an essential oil to weaner pig diets showed evidence of a reduction in *Salmonella* faecal shedding and numbers of coliforms and *Salmonella* in cecal digesta [39], and administration of an encapsulated blend of formic acid, citric acid and essential oils [40] to finishing pigs for 28 days prior to slaughter reduced *Salmonella* seroprevalence and demonstrated potential for the prevention of *Salmonella* shedding. In addition, the inclusion of cinnamaldehyde with blended organic acids and a permeabilising agent in the diets of weaned pigs experimentally infected with ETEC was found to decrease faecal *E. coli* concentrations, with no effect on the concentration of faecal lactobacilli [41]. While there is a paucity of studies involving nisin and pigs, the benefits of incorporating nisin into the diets of poultry [42] and rabbits [43] have been established.

In this study, we have provided proof of concept that the natural antimicrobial peptide nisin in combination with natural plant extracts such as trans-cinnamaldehyde could expand the spectrum of useful therapeutics and form a novel strategy in the control of ETEC infection in post-weaning pigs. However, in vivo studies along with genomic characterization and transcriptomic and proteomic profiling of the gut microbiota will be necessary to validate the antimicrobial efficacy and safety of these antimicrobial combinations for use in pigs.

## 4. Materials and Methods

### 4.1. Bacterial Strains and Growth Conditions

*Lactococcus lactis* NZ9700 (a nisin A producing strain used for peptide purification) was grown in M17 broth supplemented with 0.5% glucose (GM17) or GM17 agar at 30 °C. *E. coli* strains F150F3, F2S2, F3P3, K88F4 and F18ab were grown in Luria–Bertani (LB) broth (5 g/L yeast extract [Oxoid, Hampshire, UK], 10 g/L tryptone [Oxoid] and 10 g/L NaCl [Merck, Nottingham UK]), incubated overnight at 37 °C and shaken at 170 rpm. *E. coli* strains F150F3, F2S2 and F3P3 were isolates from pooled faecal samples from across different pig production stages and the designation F refers to Farm. Enterotoxigenic *E. coli* (ETEC) strains K88F4 and F18ab were PCR-positive for F4 and F18 adhesins, respectively. *E. coli* K88F4 was determined to be serotype O147 and positive for STb, LT and EAST1 toxins but negative for STa. *E. coli* F18ab was determined to be serotype O141 and negative for STa, STb, EAST1 and LT toxins.

### 4.2. Minimum Inhibitory Concentration Assays

Minimum inhibitory concentration (MIC) determinations were carried out in triplicate in 96-well microtitre plates (Sarstedt, Rheinbach, Germany) as described previously [36,37]. Briefly, target strains were grown overnight in the appropriate conditions and medium, subcultured into fresh broth and allowed to grow to an OD_600_ of ~0.5 and diluted to a final concentration of 10^5^ cfu mL^−1^ in a volume of 0.2 mL. Chloramphenicol, penicillin G, erythromycin, tetracycline, colistin and polymyxin B (Sigma, Steinheim, Germany) were resuspended in LB broth to a stock concentration of 128 or 256 μg/mL. The antibiotics were adjusted to 16, 32, 64 or 128 μg/mL starting concentration and 2-fold serial dilutions of each compound were made in 96-well plates for a total of 12 dilutions. The MIC assays of essential oils were carried out as above but were diluted to a starting concentration of 2 mg/mL for serial dilution of thymol, carvacrol and trans-cinnamaldehyde. Purified nisin was adjusted to a 120 µM (400 μg/mL) starting concentration and 2-fold serial dilutions were carried out. The target strain was then added and after incubation for 16 h at 37 °C, the MIC was read as the lowest concentration causing inhibition of visible growth.

### 4.3. Nisin Purification

Nisin was purified according to previously described protocols [44,45,46]. The purified nisin peptide was subjected to MALDI-ToF Mass Spectrometric analysis to confirm purity before use.

### 4.4. Growth Curve Experiments

For growth experiments, overnight cultures were transferred (10^7^ cfu mL^−1^ in a volume of 1.0 mL) into LB supplemented with the relevant concentration of nisin A and antibiotic/peptide or essential oil/peptide/EDTA combinations, and subsequently 0.2 mL was transferred to 96-well microtitre plates (Sarstedt). Cell growth was measured spectrophotometrically over 24 h or 48 h periods by using a SpectraMax M3 spectrophotometer (Molecular Devices, Sunnyvale, CA, USA).

### 4.5. Kill Assay Analysis

For kill assays, overnight cultures of target strains were transferred into LB broth (1 mL) containing nisin-purified peptide in combination with cinnamaldehyde and EDTA at the appropriate concentration. Samples were incubated for 3 h at 37 °C before serial dilution in Ringers solution followed by enumeration on LB agar plates. All experiments were carried out in triplicate.

### 4.6. Statistical Analysis

*E. coli* data were checked for normality and homogeneity of variances using the Shapiro–Wilk test and Levene’s test, respectively. All comparisons were based on the mean ± standard deviation. Parametric data were analyzed using independent *t*-tests. Non-parametric data were analyzed by the Mann–Whitney U-test. All tests were performed using a 5% level of significance. All statistically significant results are complimented with the corresponding effect size using Cohen’s d classification.

## 5. Conclusions

The increasing pressure on the livestock industry to halt the use of in-feed antibiotics has initiated new research to find safe and efficient alternatives. The combination of nisin peptides, essential oils such as cinnamaldehyde, and EDTA could pave the way for new treatment concepts when it comes to PWD, in particular towards Gram-negative bacteria including drug-resistant ETEC.

## Figures and Tables

**Table 1:** Minimum inhibitory concentration determinations of nisin A, and the essential oils thymol, carvacrol and trans-cinnamaldehyde against *E. coli* strains of swine origin. ^‡^ denotes enterotoxigenic *Escherichia coli* (ETEC) strains. Results are expressed as the mean of triplicate assays.

Antibiotic	*E. coli* Strain				
	F150F3	F2S2	K88F4 ^‡^	F3P3	F18ab ^‡^
Chloramphenicol	>50	12.5	>50	>50	12.5
Tetracycline	>50	1.56	50	25	50
Penicillin G	>50	25	25	25	12.5
Streptomycin	>50	25	>50	12.5	1.56
Cefoxitin	>50	>50	>50	>50	>50
Erythromycin	>50	>50	>50	>50	50
Lincomycin	>50	>50	>50	>50	>50
Ceftazidine	<0.4	<0.4	<0.4	<0.4	<0.4
Cefuroxime	6.25	3.12	6.25	6.25	6.25
Cefradine	25	12.5	25	25	12.5
Cefsulodin	>50	50	50	50	50
Colistin	0.39	0.39	<0.2	<0.2	25
Polymyxin B	0.39	0.39	<0.2	<0.2	25
Carvacrol	>1250	>1250	>1250	>1250	>1250
Cinnamaldehyde	1250	1250	1250	>1250	1250
Thymol	>1250	>1250	>1250	>1250	>1250
Nisin	200	200	200	200	200
